# Associations between Chronic Kidney Disease and Thinning of Neuroretinal Layers in Multiethnic Asian and White Populations

**DOI:** 10.1016/j.xops.2023.100353

**Published:** 2023-06-20

**Authors:** Shivani Majithia, Crystal Chun Yuen Chong, Miao Li Chee, Marco Yu, Zhi Da Soh, Sahil Thakur, Raghavan Lavanya, Tyler Hyungtaek Rim, Simon Nusinovici, Victor Koh, Charumathi Sabanayagam, Ching-Yu Cheng, Yih-Chung Tham

**Affiliations:** 1 Singapore Eye Research Institute, Singapore National Eye Centre, Singapore; 2 Ophthalmology & Visual Sciences Academic Clinical Program (Eye ACP), Duke-NUS Medical School, Singapore; 3 Centre for Innovation & Precision Eye Health, Department of Ophthalmology, Yong Loo Lin School of Medicine, National University of Singapore, Singapore; 4 Department of Ophthalmology, National University Hospital, Singapore

**Keywords:** Asian, Chronic kidney disease, Ganglion cell-inner plexiform layer, Retinal nerve fiber layer, White

## Abstract

**Purpose:**

To evaluate the relationships between chronic kidney disease (CKD) with retinal nerve fiber layer (RNFL) and ganglion cell-inner plexiform layer (GCIPL) thickness profiles of eyes in Asian and White populations.

**Design:**

Cross-sectional analysis.

**Participants:**

A total of 5066 Asian participants (1367 Malays, 1772 Indians, and 1927 Chinese) from the Singapore Epidemiology of Eye Diseases Study (SEED) were included, consisting of 9594 eyes for peripapillary RNFL analysis and 8661 eyes for GCIPL analysis. Additionally, 45 064 White participants (87 649 eyes) from the United Kingdom Biobank (UKBB) were included for both macular RNFL analysis and GCIPL analysis.

**Methods:**

Nonglaucoma participants aged ≥ 40 years with complete data for estimated glomerular filtration rate (eGFR) were included from both SEED and UKBB. In SEED, peripapillary RNFL and GCIPL thickness were measured by Cirrus HD-OCT 4000. In UKBB, macular RNFL and GCIPL were measured by Topcon 3D-OCT 1000 Mark II. Chronic kidney disease was defined as eGFR < 60 ml/min/1.73 m^2^ in both data sets. To evaluate the associations between kidney function status with RNFL and GCIPL thickness profiles, multivariable linear regression with generalized estimating equation models were performed in SEED and UKBB data sets separately.

**Main Outcome Measures:**

Average peripapillary and macular RNFL thickness and macular GCIPL thickness.

**Results:**

In SEED, after adjusting for age, gender, ethnicity, systolic blood pressure, antihypertensive medication, diabetes, hyperlipidemia, body mass index, smoking status, and intraocular pressure, presence of CKD (β = −1.31; 95% confidence interval [CI], −2.37 to −0.26; *P* = 0.015) and reduced eGFR (per 10 ml/min/1.73 m^2^; β = −0.32; 95% CI, −0.50 to −0.13; *P* = 0.001) were associated with thinner average peripapillary RNFL. Presence of CKD (β = −1.63; 95% CI, −2.42 to −0.84) and reduced eGFR (per 10 ml/min/1.73 m^2^; β = −0.30; 95% CI, −0.44 to −0.16) were consistently associated with thinner GCIPL in SEED (all *P* < 0.001). In UKBB, after adjusting for the above-mentioned covariates (except ethnicity), reduced eGFR (per 10 ml/min/1.73 m^2^; β = −0.06; 95% CI, −0.10 to −0.01; *P* = 0.008) was associated with thinner macular RNFL and CKD (β = −0.62; 95% CI, −1.16 to −0.08; *P* = 0.024) was associated with thinner average GCIPL.

**Conclusion:**

We consistently observed associations between CKD and thinning of RNFL and GCIPL across Asian and White populations' eyes. These findings further suggest that compromised kidney function is associated with RNFL and GCIPL thinning.

**Financial Disclosure(s):**

The author(s) have no proprietary or commercial interest in any materials discussed in this article.

Glaucoma and chronic kidney disease (CKD) are both chronic, age-related diseases that are major global health concerns.^1^, ^2^, ^3^ The burden of glaucoma and CKD is expected to increase, due to a rapidly aging population.^2^^,^^3^ Although previous reports indicated that patients with a history of CKD had increased glaucoma risk,^4^, ^5^, ^6^ the underlying explanation for this association is still unclear.

Measurements of the retinal nerve fiber layer (RNFL) and ganglion cell-inner plexiform layer (GCPIL) are routinely performed using OCT imaging to detect, diagnose, and monitor glaucoma. Previous studies have investigated the relationship of CKD with RNFL and GCIPL thickness profiles but presented inconsistent findings. This was partly because of small sample sizes and confinement to specific populations in these previous studies.^7^, ^8^, ^9^, ^10^ For example, a study in Taiwan with 171 CKD cases and 40 controls observed thinner RNFL and GCIPL in participants with CKD.^7^ However, another study with White participants observed no difference in RNFL thickness between participants with CKD and controls.^8^ Furthermore, most other studies only examined participants undergoing hemodialysis for end-stage CKD.^11^, ^12^, ^13^

To better elucidate this aspect, a comprehensive evaluation on the associations between CKD with RNFL and GCIPL thickness in a larger sample size is warranted. Hence, this study aimed to evaluate the relationships between CKD and RNFL and GCIPL thickness profiles across Asian and White populations.

## Methods

### Study Populations

#### SEED

We conducted a cross-sectional population-based study from study subjects enrolled in the 6-year follow-up visit from the Singapore Epidemiology Eye Disease study (SEED), comprised of 3 major Asian ethnic groups: Malays, Indians, and Chinese. Details of the SEED study’s methodology were reported previously.^14^ Briefly, the study randomly sampled adults aged ≥ 40 years from the southwestern part of Singapore. At baseline, 10 033 subjects participated and were followed up on a 6-year interval. At the 6-year follow-up visits, the study sample comprised of 1901 Malays (year 2011–2013; response rate, 72.1%), 2200 Indians (year 2013–2015; response rate, 75.5%), and 2661 Chinese (year 2015–2017; response rate, 87.7%).

### United Kingdom Biobank

The United Kingdom Biobank (UKBB) is a large, multisite, population-based study comprised of > 500 000 UK residents aged 40–69 years at baseline (2006–2010).^15^ Physical measurements and health questionnaire data were collected from 22 assessment centers across the UK. Ocular assessments and history of eye diseases from self-administered questionnaires were added in 2009 across 6 assessment centers in the UK. Data were available for 132 041 participants.^16^ Details about the UKBB can be found at their website (www.ukbibobank.ac.uk).

Informed consent was obtained from all study participants for both SEED and UKBB. All study procedures for SEED and the UKBB were conducted in accordance with the Declaration of Helsinki. The SEED study was approved by the SingHealth Centralized Institutional Review Board, while the UKBB study was approved by the National Information Governance Board for Health and Social Care and National Health Service North West Multi-Centre Research Ethics Committee.

### Inclusion and Exclusion Criteria

Participants from SEED and the UKBB that underwent OCT imaging and had complete estimated glomerular filtration rate (eGFR) data were included. Additionally, participants aged ≥ 40 years with relevant systemic, ocular, and lifestyle-related data, including history of diabetes, hypertension, hyperlipidemia, smoking status, body mass index (BMI), and intraocular pressure (IOP) were included. Study eyes with poor OCT image quality were excluded from analysis. Additionally, confirmed glaucoma cases in SEED (diagnosed based on the International Society for Geographical and Epidemiological Ophthalmology guidelines) and the UKBB (based on International Classification of Diseases code for glaucoma or self-reported history of glaucoma or glaucoma surgery/laser) were also excluded.

### Ophthalmic Assessment

#### SEED

All participants underwent a standardized ocular examination at the Singapore Eye Research Institute. Before pupil dilation, IOP was measured using a Goldmann applanation tonometer (Haag-Streit). Additionally, gonioscopy and 24-2 SITA Fast Humphrey visual field (Humphrey Field Analyzer II; Humphrey Instruments) test were performed for glaucoma suspects and participants with known history of glaucoma before dilation. After dilation with tropicamide 1% and phenylephrine 2.5%, fundus examinations were performed.

### UKBB

Intraocular pressure was measured using the Ocular Response Analyzer noncontact tonometer (Reichert, Philadelphia), and the Goldmann-correlated IOP values were used in this analysis.

### Spectral-Domain–OCT Imaging

#### SEED

After pupil dilation, images were acquired using a commercially available spectral-domain OCT (SD-OCT) (Cirrus 4000 HD-OCT; Carl Zeiss Meditec, Inc.). Optic disc cube (200 × 200) and macular cube (512 × 128) scans were acquired, covering a measurement area of 6 × 6mm^2^. Retinal nerve fiber layer and GCIPL thickness parameters were determined by an automated algorithm incorporated in the Cirrus HD-OCT review software. Detailed descriptions of the measurement algorithms have been described previously.^17^^,^^18^ In brief, for peripapillary RNFL measurement, the algorithm automatically identified Bruch’s membrane opening as the disc area, and the reference plane was determined 200 μm above the level of Bruch’s membrane plane. Additionally, for GCIPL measurement, the algorithm measured the GCIPL thickness inside a 14.13 mm^2^ elliptical annulus area centered on the fovea. Superior and inferior GCIPL hemisphere thicknesses were averaged from superior and inferior subfields, respectively. Poor scan quality was defined as poor OCT signal strength (< 6), misaligned scans, motion/blinking artifacts, segmentation errors, and retinal diseases diagnosed or observed from OCT scans known to impact GCIPL and RNFL thickness (macular edema, age-related macular degeneration, diabetic retinopathy, and other retinopathy cases).

### UKBB

A subset of the cohort (67 321 participants) had macular SD-OCT imaging collected. Certified UKBB ophthalmic technicians who were trained in collaboration with the Moorfields Eye Hospital Reading center captured SD-OCT images using the Topcon 3D OCT 1000 Mark II (Topcon).^19^ Macular scans for both eyes were acquired using the 3D macular volume scan protocol (raster scan of 512 A-scans by 128 B-scans covering a 6 × 6 mm^2^ area) in a dark room without pupil dilation.^16^^,^^19^ We included macular RNFL and GCIPL thickness in our final analysis. The peripapillary RNFL thickness measurement was not available in the UKBB data set.

A validated custom imaging segmentation software (version 1.6.1.1) by the Topcon Advanced Biomedical Imaging Laboratory was used to obtain automated segmentation of retinal boundaries and inner layers.^20^ Based on these segmentations, macular RNFL and GCIPL thicknesses were measured. Scans with poor OCT signal strength (< 45), segmentation error and blinking/motion artifacts were excluded.^16^^,^^21^ We further excluded scans of participants with diagnosed and/or self-reported ocular and systemic diseases known to impact GCIPL and RNFL thickness; these included neurodegenerative diseases, age-related macular degeneration, and glaucoma.

### Systemic Examinations and Other Measurements

Venous blood samples were collected in SEED and UKBB for biochemical testing. Body mass index was calculated as body weight (in kilograms) divided by body height (in meters) squared.

Diabetes was defined by random glucose ≥ 11.1 mmol/L, glycosylated hemoglobin ≥ 6.5%, use of diabetic medication(s) and/or self-reported history in SEED. On the other hand, in UKBB, diabetes was defined based on the International Classification of Diseases code for diabetes or self-reported history of diabetes. In both data sets, hyperlipidemia was defined as total cholesterol ≥ 6.2 mmol/L, use of lipid lowering drugs, or self-reported history of hyperlipidemia. In both studies, hypertension was defined by systolic blood pressure (BP) ≥ 140 mmHg, diastolic BP ≥ 90 mmHg, use of hypertensive medication, and/or self-reported history of hypertension for both studies.

Lastly, a detailed interviewer-administered questionnaire was used to obtain participant information regarding medication use, systemic and ocular history, sociodemographic information, and current smoking status for each respective study.

### Measurement of eGFR and Definition of CKD

Kidney function was indicated by the calculation of eGFR. Lower eGFR is indicative of poorer kidney function. Estimated glomerular filtration rate was calculated from the measurement of serum creatinine, using the Chronic Kidney Disease Epidemiology Collaboration equation as follows:^22^

$$GFR=141\times {\left(\min\left(\frac{creatinine\left(mg/dl\right)}{k},1\right)\right)}^{a}\times {\left(\max\left(\frac{creatinine\left(mg/dl\right)}{k},1\right)\right)}^{-1.209}\times 0.993^{age}\times 1.018^{c}$$

where, a = −0.411, k = 0.9, c = 0 for males; and a = −0.329, k = 0.7, c = 1 for females; min indicates the lower value between serum creatinine/k or 1, and max indicates the higher value between serum creatinine/k or 1.

In both data sets, we defined CKD as eGFR < 60 ml/min/1.73 m^2^. We further stratified kidney function status into normal (eGFR > 90 ml/min/1.73 m^2^), mild (eGFR 60–89 ml/min/1.73 m^2^), moderate (eGFR 45–59 ml/min/1.73 m^2^), and severe decline (eGFR < 45 ml/min/1.73 m^2^).

### Statistical Analysis

All statistical analyses were performed using R language (R version 3.5.3, R Foundation for Statistical Computing). Age, systolic BP, BMI, IOP, and eGFR were analyzed as continuous variables, whereas gender, ethnicity, antihypertensive medication, diabetes, hyperlipidemia, smoking status, CKD, and stages of kidney function were analyzed as categorical variables.

To investigate the associations between kidney function status with RNFL and GCIPL thickness profiles, we performed multivariable linear regression models with generalized estimating equations method (exchangeable correlation structures and a Gaussian link) in SEED and UKBB data sets separately. Additionally, the associations of CKD, decrease in eGFR, and stages of kidney function with RNFL and GCIPL were evaluated in separate multivariable models. In the models, we adjusted for potential confounders, including age, gender, ethnicity (except in UKBB), systolic BP, antihypertensive medication, diabetes, hyperlipidemia, BMI, smoking status, and IOP. The *P* value for significance was set at < 0.05.

## Results

**Figure 1:**
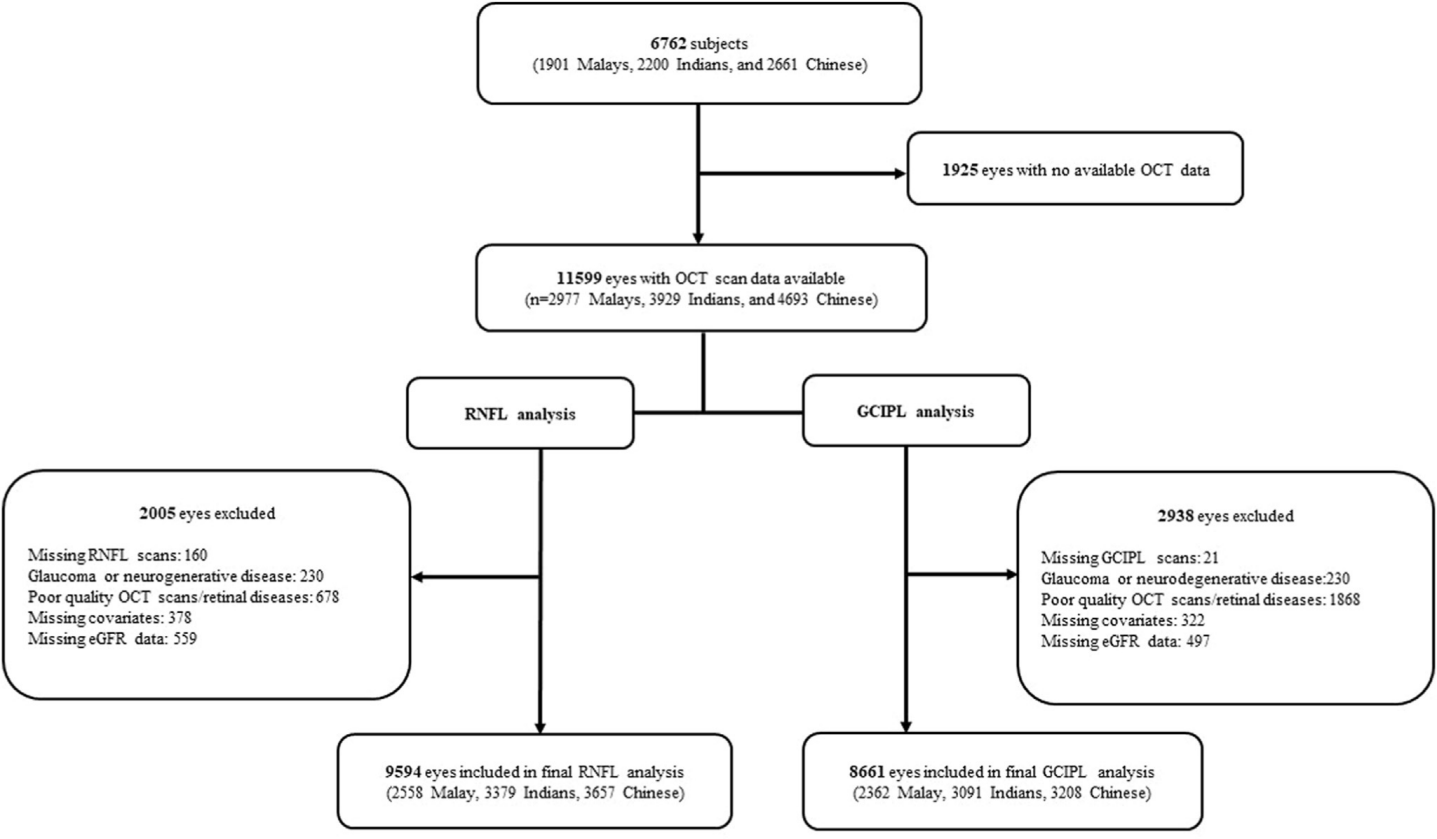
Flow chart of final study participants included for the Singapore Epidemiology of Eye Diseases Study. eGFR = estimated glomerular filtration rate; GCIPL = ganglion cell-inner plexiform layer; RNFL = retinal nerve fiber layer.

**Figure 2:**
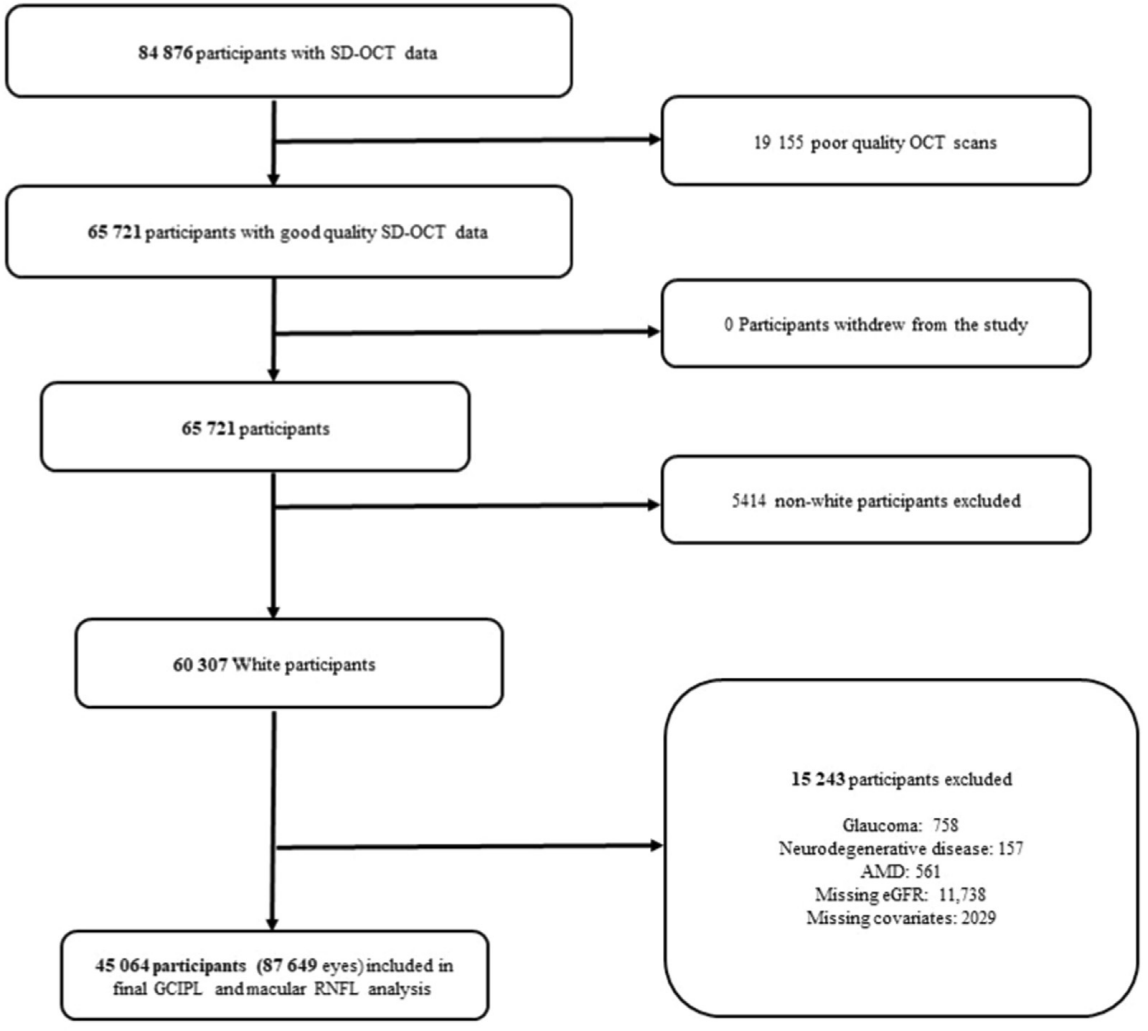
Flow chart of final study participants included for United Kingdom Biobank. AMD = age-related macular degeneration; eGFR = estimated glomerular filtration rate; GCIPL = ganglion cell-inner plexiform layer; RNFL = retinal nerve fiber layer; SD-OCT = spectral-domain OCT.

Of the original 6762 participants (13 524 eyes) in SEED, 1925 eyes were excluded due to unavailable OCT data. After the exclusion of poor-quality scans and retinal diseases that affect RNFL and GCIPL thickness, 9594 eyes were included for final peripapillary RNFL analysis, and 8661 eyes were included for GCIPL analysis (Fig 1). The mean age of participants in SEED was 61.5 ± 8.5 years, 51.3% of participants were female, 9.6% of participants had CKD, 29.2% had diabetes, and 65.5% had hypertension (Table 1). Characteristics of SEED participants by ethnicity are detailed in Table S2 (available at www.ophthalmologyscience.org). In UKBB, 84 876 participants had OCT data available. After the exclusion of poor-quality scans, non-White participants and retinal diseases that affect macular RNFL and GCIPL thickness measurements, 87 649 eyes were included for final macular RNFL and GCIPL analyses (Fig 2). The mean age of UKBB participants was 56.8 ± 8 years, 54.6% of the participants were female, 2.2% of the participants had CKD, 4.6% had diabetes, and 55.7% had hypertension (Table 1).

**Table 1:** Characteristics of Included Participants

	SEED	UKBB
	Overall (N = 5066)	White (n = 45 064)
Demographic and systemic characteristics		
Age, yrs	61.5 (8.5)	56.8 (8)
Gender, female, n (%)	2598 (51.3)	24 622 (54.6)
Ethnicity		NA
Malay	1367 (27.0)	
Indian	1772 (35.0)	
Chinese	1927 (38.0)	
Positive history of CKD, n (%)	488 (9.6)	996 (2.2)
eGFR, ml/min/1.73 m^2^	85.7 (18.2)	89.8 (13)
Stages of kidney function (based on eGFR), n (%)		
≥ 90 ml/min/1.73 m^2^	2545 (50.2)	25 203 (55.9)
60–89 ml/min/1.73 m^2^	2033 (40.1)	18 865 (41.9)
45–59 ml/min/1.73 m^2^	306 (6.0)	864 (1.9)
< 45 ml/min/1.73 m^2^	182 (3.6)	132 (0.3)
Diabetes, n (%)	1478 (29.2)	2072 (4.6)
Hypertension, n (%)	3318 (65.5)	25 115 (55.7)
Hyperlipidemia, n (%)	2956 (58.4)	21 861 (48.5)
BMI, kg/m^2^	25.5 (4.6)	27.2 (4.7)
Current smoking status, n (%)	680 (13.4)	4331 (9.6)
IOP, mmHg	14.8 (2.9)	15.8 (3.9)
RNFL thickness, μm∗	91.5 (11.4)	28.6 (6.2)
Macular GCIPL thickness, μm	79.1 (8.6)	74.1 (8.4)

BMI = body mass index; CKD = chronic kidney disease; eGFR = estimated glomerular filtration rate; GCIPL = ganglion cell-inner plexiform layer; IOP = intraocular pressure; RNFL = retinal nerve fiber layer; SEED = Singapore Epidemiology of Eye Diseases Study; UKBB = United Kingdom Biobank. Data presented are mean (standard deviation) or frequency (%), where appropriate.

∗ Peripapillary RNFL thickness measurement in SEED and macular RNFL thickness measurement in UKBB.

The associations between kidney function and peripapillary RNFL in SEED are described in Table 3. After adjusting for age, gender, ethnicity, systolic BP, antihypertensive medication, diabetes, hyperlipidemia, BMI, smoking status, and IOP, CKD was associated with thinner average RNFL (β = −1.31; 95% confidence interval [CI], −2.37 to −0.26; *P* = 0.015) and inferior quadrant RNFL (β = −2.59; 95% CI, −4.41 to −0.77; *P* = 0.005). Reduced eGFR (per 10 ml/min/1.73 m^2^) was significantly associated with thinner average RNFL (β = −0.32; 95% CI, −0.50 to −0.13; *P* = 0.001), superior quadrant RNFL (β = −0.37; 95% CI, −0.67 to −0.08; *P* = 0.012), and inferior quadrant RNFL (β = −0.59; 95% CI, −0.91 to −0.27; *P* < 0.001). When categorized by CKD stages, severe kidney function decline (eGFR < 45 ml/min/1.73 m^2^) was significantly associated with thinner average RNFL (β = −2.86; 95% CI, −4.50 to −1.22; *P* < 0.001), superior quadrant RNFL (β = −3.72; 95% CI, −6.20 to −1.24; *P* = 0.003) and inferior quadrant RNFL (β = −6.07; 95% CI, −8.84 to −3.30; *P* < 0.001). Additionally, when evaluating these associations by Asian ethnicity in SEED, we observed more prominent associations between kidney function and RNFL thinning in Malay and Indian eyes (Table S4, available at www.ophthalmologyscience.org).

**Table 3:** Associations between CKD and Kidney Function with Peripapillary RNFL Thickness in SEED

	Number of Eyes	Average RNFL (μm)		Superior RNFL (μm)		Inferior RNFL (μm)	
		β (95% CI)†	*P* Value	β (95% CI)†	*P* Value	β (95% CI)†	*P* Value
No CKD	8726	Ref		Ref		Ref	
Presence of CKD∗	868	−1.31 (−2.37 to −0.26)	0.015	−0.98 (−2.63 to 0.67)	0.244	−2.59 (−4.41 to −0.77)	0.005
eGFR (per 10 ml/min/1.73m^2^ decrease)	9594	−0.32 (−0.50 to −0.13)	0.001	−0.37 (−0.67 to −0.08)	0.012	−0.59 (−0.91 to −0.27)	< 0.001
Stages of kidney function (based on eGFR):							
≥ 90 ml/min/1.73 m^2^	4915	Ref		Ref			
60–89 ml/min/1.73 m^2^	3811	−0.17 (−0.84 to 0.49)	0.609	−0.19 (−1.20 to 0.82)	0.716	−0.21 (−1.34 to 0.92)	0.714
45–59 ml/min/1.73 m^2^	554	−0.62 (−1.95 to 0.72)	0.366	0.38 (−1.75 to 2.50)	0.728	−0.83 (−3.13 to 1.48)	0.480
<45 ml/min/1.73 m^2^	314	−2.86 (−4.50 to −1.22)	< 0.001	−3.72 (−6.20 to −1.24)	0.003	−6.07 (−8.84 to −3.30)	< 0.001
*P* trend			0.008		0.079		0.002

CI = confidence interval; CKD = chronic kidney disease; eGFR = estimated glomerular filtration rate; RNFL = retinal nerve fiber layer.

∗ Defined as eGFR < 60 ml/min/1.73m^2^.

† Adjusted for age, gender, ethnicity, systolic blood pressure, antihypertensive medication, diabetes, hyperlipidemia, body mass index, smoking status, and intraocular pressure

Table 5 shows the associations between kidney function and macular GCIPL thickness in SEED. After adjustments for age, gender, ethnicity, systolic BP, antihypertensive medication, diabetes, hyperlipidemia, BMI, smoking status, and IOP, CKD was associated with thinner average GCIPL (β = −1.63; 95% CI, −2.42 to −0.84), superior hemisphere GCIPL (β = −1.49; 95% CI, −2.28 to −0.69), and inferior hemisphere GCIPL (β = −1.77; 95% CI, −2.61 to −0.92) (all *P* < 0.001). Similarly, reduced eGFR (per 10 ml/min/1.73 m^2^) was associated with thinner average GCIPL (β = −0.30; 95% CI, −0.44 to −0.16), superior hemisphere GCIPL (β = −0.29; 95% CI, −0.43 to −0.15) and inferior hemisphere GCIPL (β = −0.31; 95% CI, −0.45 to −0.16) (all *P* < 0.001). When categorized by CKD stages, mild (eGFR 60–89 ml/min/1.73 m^2^), moderate (eGFR 45–59 ml/min/1.73 m^2^), and severe (eGFR < 45 ml/min/1.73 m^2^) kidney function decline were significantly associated with thinner average, superior hemisphere, and inferior hemisphere GCIPL (all *P* ≤ 0.040). Additionally, when evaluating these associations by Asian ethnicity in SEED, more prominent associations were observed in Malay and Indian eyes. (Table S6, available at www.ophthalmologyscience.org)**.**

**Table 5:** Associations between CKD and Kidney Function with Macular GCIPL Thickness in SEED

	Number of Eyes	Average GCIPL (μm)		Superior Hemisphere GCIPL (μm)		Inferior Hemisphere GCIPL (μm)	
		β (95% CI)†	*P* Value	β (95% CI)†	*P* Value	β (95% CI)†	*P* Value
No CKD	7980	Ref		Ref		Ref	
Presence of CKD∗	681	−1.63 (−2.42 to −0.84)	< 0.001	−1.49 (−2.28 to −0.69)	< 0.001	−1.77 (−2.61 to −0.92)	< 0.001
eGFR (Per 10 ml/min/1.73m^2^ decrease)	8661	−0.30 (−0.44 to −0.16)	< 0.001	−0.29 (−0.43 to −0.15)	< 0.001	−0.31 (−0.45 to −0.16)	< 0.001
Stages of kidney function (based on eGFR)							
≥ 90 ml/min/1.73 m^2^	4557	Ref		Ref		Ref	
60–89 ml/min/1.73 m^2^	3423	−0.47 (−0.90 to −0.04)	0.032	−0.47 (−0.90 to −0.03)	0.036	−0.47 (−0.91 to −0.02)	0.040
45–59 ml/min/1.73 m^2^	440	−1.38 (−2.37 to −0.38)	0.007	−1.28 (−2.26 to −0.30)	0.010	−1.45 (−2.51 to −0.39)	0.007
< 45 ml/min/1.73 m^2^	241	−3.00 (−4.26 to −1.75)	< 0.001	−2.76 (−4.08 to −1.45)	< 0.001	−3.23 (−4.53 to −1.93)	< 0.001
*P* trend			< 0.001		< 0.001		< 0.001

CI = confidence interval; CKD = chronic kidney disease; eGFR = estimated glomerular filtration rate; GCIPL = ganglion cell-inner plexiform layer.

∗ Defined as eGFR < 60 ml/min/1.73m^2^.

† Adjusted for age, gender, ethnicity, systolic blood pressure, antihypertensive medication, diabetes, hyperlipidemia, body mass index, smoking status, and intraocular pressure.

Using White data from the UKBB, we further evaluated the associations between kidney function with average macular RNFL and GCIPL thickness (Table 7). After adjusting for age, gender, systolic BP, antihypertensive medication, diabetes, hyperlipidemia, BMI, smoking status, and IOP, reduced eGFR (per 10 ml/min/1.73 m^2^) was associated with average macular RNFL thinning (β = −0.06; 95% CI, −0.10 to −0.01; *P* = 0.008). When categorized by CKD stages, mild (eGFR 60–89 ml/min/1.73 m^2^) kidney function decline was associated with average macular RNFL thinning (β = −0.10; 95% CI, −0.20 to 0.00; *P* = 0.045). Broadly, increased severity of kidney disease was also associated with average macular RNFL thinning (*P* trend = 0.006). Furthermore, CKD was associated with average GCIPL thinning (β = −0.62; 95% CI, −1.16 to −0.08; *P* = 0.024). In particular, severe kidney function decline (eGFR < 45 ml/min/1.73 m^2^) was associated with average GCIPL thinning (β = −1.34; 95% CI, −2.40 to −0.28; *P* = 0.014).

**Table 7:** Associations between CKD and Kidney Function with Macular RNFL and GCIPL Thickness in United Kingdom Biobank

	Number of Eyes	Average Macular RNFL (μm)		Number of Eyes	Average GCIPL (μm)	
		β (95% CI)†	*P* Value		β (95% CI)†	*P* Value
No CKD	85 725	Ref		85 725	Ref	
Presence of CKD∗	1924	−0.36 (−0.74 to 0.02)	0.065	1 924	−0.62 (−1.16 to −0.08)	0.024
eGFR (Per 10 ml/min/1.73m^2^ decrease)	87 649	−0.06 (−0.10 to −0.01)	0.008	87 649	−0.06 (−0.12 to 0.00)	0.069
Stages of kidney function (based on eGFR)						
≥ 90 ml/min/1.73 m^2^	49 067	Ref		49 067	Ref	
60–89 ml/min/1.73 m^2^	36 658	−0.10 (−0.20 to 0.00)	0.045	36 658	−0.11 (−0.25 to 0.04)	0.157
45–59 ml/min/1.73 m^2^	1666	−0.35 (−0.77 to 0.06)	0.097	1666	−0.58 (−1.18 to 0.02)	0.060
< 45 ml/min/1.73 m^2^	258	−0.81 (−1.64 to 0.02)	0.057	258	−1.34 (−2.40 to −0.28)	0.014
*P* trend			0.006			0.015

CI = confidence interval; CKD = chronic kidney disease; eGFR = estimated glomerular filtration rate; GCIPL = ganglion cell-inner plexiform layer; RNFL = retinal nerve fiber layer.

∗ Defined as eGFR < 60 ml/min/1.73m^2^.

† Adjusted for age, gender, systolic blood pressure, antihypertensive medication, diabetes, hyperlipidemia, body mass index, smoking status and intraocular pressure.

## Discussion

We investigated the associations of kidney function status with RNFL and GCIPL thickness in a multiethnic Asian population study. We also performed validation in UKBB’s White data set. Leveraging on approximately 9500 Asians patients' eyes and 87 000 White patients' eyes, this is one of the largest studies to date elucidating this association. In individuals with CKD and suboptimal kidney function, we observed significant RNFL and GCIPL thinning. This finding was largely consistent across Asian and White eyes. Our findings further support the notion that individuals with CKD may be more susceptible to RNFL and GCIPL thinning, which is an important marker for glaucoma development.

In Asian patients' eyes, CKD and reduced eGFR were associated with thinner peripapillary RNFL. Similarly, in White patients' eyes, reduced eGFR was associated with thinner macular RNFL. Consistently, in Asian patients' eyes, we observed that CKD and reduced eGFR were associated with thinner GCIPL. Chronic kidney disease was also associated with GCIPL thinning in White patients' eyes. Across Asian and White patients' eyes, when evaluating the different stages of kidney disease, we observed a significant trend between declining kidney function with thinner RNFL and GCIPL. Taken together, these findings across Asian and White eyes further corroborate the overall relationship of kidney function with RNFL and GCIPL thickness.

Previous studies have reported on the relationship of CKD with RNFL and GCIPL thickness. Similar to our study, Wu et al^7^ noted that Taiwanese CKD patients had a reduced average RNFL and ganglion cell complex thickness. Furthermore, the LIFE-adult study (a large German population–based study) observed that thinner circumpapillary RNFL was associated with poor renal function.^23^ Additionally, similar to our UKBB findings, an Irish study with a smaller sample size of 223 participants observed that a thinner ganglion cell layer and inner plexiform layer were only associated with severe stages of CKD.^10^ Altogether, these results suggest that compromised kidney function is linked to damage in both the RNFL and GCIPL.

The association between kidney disease and neuroretinal thinning may be explained by shared structural and pathophysiologic mechanisms that affect both the kidney and retina.^24^^,^^25^ Structurally, the retina and kidney are both highly vascularized organs that are vulnerable to microvascular damage from systemic diseases.^4^^,^^26^ Additionally, pathogenic mechanisms, such as chronic inflammation and oxidative stress, have been known to cause injury to retinal and renal layers.^4^^,^^24^^,^^27^ Furthermore, the renin-angiotensin system is found locally in the retina and kidneys.^28^^,^^29^ The renin-angiotensin system is a hormone system that plays a critical role in regulating BP and fluid balance in the body.^28^ As a result, dysregulation of renin-angiotensin system may also lead to inflammation and oxidative stress impacting the pathogenesis and progression of both kidney and retinal diseases.^24^^,^^29^^,^^30^ Therefore, retinal irregularities, such as RNFL and GCIPL thinning, may be explained by the occurrence of retinal microvascular damage caused by renal dysfunction.^31^^,^^32^ Furthermore, given that RNFL and GCIPL thickness are established markers of glaucoma,^33^, ^34^, ^35^ it is advisable for clinicians to closely observe the pattern of RNFL and GCIPL loss as a means of distinguishing between glaucomatous and CKD-related neuroretinal loss.^36^ On this note, individuals with CKD may warrant regular eye examinations.

Interestingly, when evaluating the associations between kidney function with RNFL and GCIPL thickness by ethnicity in Asian eyes, we observed that RNFL and GCIPL thinning were more prominently observed in Malay and Indian eyes (Tables S4, S6, available at www.ophthalmologyscience.org). This observation could be partially explained by the inherent thinner RNFL and GCIPL profiles in Indians and Malay eyes,^37^^,^^38^ thus potentially predisposing them to be more susceptible to retinal microvasculature damage caused by CKD.

The strengths of this study include its large sample with validation of findings across Asians and Whites through the use of SEED and UKBB data sets. In addition, we performed a robust analysis which accounted for a wide range of potential confounders. Furthermore, a stringent quality check for OCT scans was performed, and scans with poor signal strength and/or segmentation errors were excluded from final analysis. However, our study has a few limitations. First, due to the cross-sectional nature of our study, we cannot definitively infer a causal relationship between CKD with RNFL and GCIPL thickness. Therefore, future longitudinal studies are still warranted. Second, peripapillary RNFL measurement was not available in the UKBB study; nevertheless, we used macular RNFL thickness as a proxy for peripapillary RNFL. However, because macular RNFL is inherently thinner than peripapillary RNFL, this aspect of our findings ought to be evaluated with caution. Lastly, despite following a standardized statistical analysis protocol across the SEED and UKBB studies, there are differences in study methodologies between the 2 studies. Hence, future multiethnic studies with a standardized examination protocol are still warranted to further elucidate the relationship between CKD and neuroretinal layers.

In conclusion, we observed that CKD and compromised kidney function are associated with thinner RNFL and GCIPL in both Asian and White eyes. Hence, because RNFL and GCIPL are established markers of glaucoma, clinicians should closely monitor the pattern of RNFL and GCIPL loss to differentiate between neuroretinal damage caused by glaucoma and that associated with CKD.
