# Supplementary material for: Associations between Chronic Kidney Disease and Thinning of Neuroretinal Layers in Multiethnic Asian and White Populations
Source: Ophthalmol Sci. 2023 Jun 20;4(1):100353. doi: 10.1016/j.xops.2023.100353 (PMC10587624; doi:10.1016/j.xops.2023.100353)
Supplement: Table S2 [file mmc1.pdf]

Supplementary Table 1. Characteristics of Included Participants in SEED by Ethnicity

Singapore Epidemiology of Eye Diseases Study			
	**Malay (n = 1367)**	**Indian (n= 1772)**	**Chinese (n= 1927)**
**Demographic and systemic characteristics**			
**Age (years)**	61.3 (9.1)	60.9 (8.1)	62.3 (8.2)
**Gender, Female, n (%)**	737 (53.9)	876 (49.4)	985 (51.1)
**Positive history of CKD, n (%)**	215 (15.7)	137 (7.7)	136 (7.1)
**eGFR, mL/min/1.73m^2**	82.0 (21.8)	87.0 (16.6)	87.1 (16.3)
**Stages of kidney function (based on eGFR), n (%):**			
≥90 ml/min/1.73m^2	602 (44.0)	932 (52.6)	1011 (52.5)
60 to 89 ml/min/1.73m^2	550 (40.3)	703 (39.7)	780 (40.5)
45 to 59 ml/min/1.73m^2	120 (8.8)	92 (5.2)	94 (4.9)
<45 mL/min/1.73 m^2	95 (6.9)	45 (2.5)	42 (2.2)
**Diabetes, n (%)**	435 (31.8)	715 (40.4)	328 (17.0)
**Hypertension, n (%)**	963 (70.4)	1150 (64.9)	1205 (62.5)
**Hyperlipidemia, n (%)**	771 (56.4)	1099 (62.0)	1086 (56.4)
**BMI, kg/m^2**	27.0 (5.1)	26.5 (4.5)	23.7 (3.6)
**Current smoking status, n (%)**	246 (18.0)	229 (12.9)	205 (10.6)
**IOP, mmHg**	14.5 (3.1)	15.3 (2.8)	14.6 (2.7)
**Peripapillary RNFL thickness, µm**	94.6 (10.8)	87.3 (10.7)	93.2 (11.4)
**Macular GCIPL thickness, µm**	80.4 (8.5)	77.5 (8.3)	79.7 (8.8)

Abbreviations: BMI: body mass index; CKD: chronic kidney disease; eGFR: estimated glomerular filtration rate; GCIPL: ganglion cell-inner plexiform layer; IOP: intraocular pressure; RNFL: retinal nerve fiber layer

Data presented are mean (standard deviation) or frequency (%), where appropriate.
