# Supplementary material for: Associations between Chronic Kidney Disease and Thinning of Neuroretinal Layers in Multiethnic Asian and White Populations
Source: Ophthalmol Sci. 2023 Jun 20;4(1):100353. doi: 10.1016/j.xops.2023.100353 (PMC10587624; doi:10.1016/j.xops.2023.100353)
Supplement: Table S4 [file mmc2.pdf]

Supplementary Table 2. Associations between Chronic Kidney Disease and Kidney Function with RNFL thickness by Ethnicity in SEED

	Average RNFL Thickness (µm)								
	Malay			Indian			Chinese		
	**No. of eyes**	**Beta (95% CI) ***	**P value**	**No. of eyes**	**Beta (95% CI)***	**P value**	**No. of eyes**	**Beta (95% CI)***	**P value**
**No CKD**	2177	Ref		3139	Ref		3410	Ref	
**Presence of CKD†**	381	-1.19 (-2.82 to 0.44)	0.152	240	-1.82 (-3.86 to 0.22)	0.080	247	-0.53 (-2.52 to 1.45)	0.598
**eGFR (Per 10 ml/min/ $1.73m^2$ decrease)**	2558	-0.41 (-0.72 to -0.10)	**0.010**	3379	-0.56 (-0.91 to -0.21)	**0.002**	3657	0.10 (-0.24 to 0.44)	0.559
**Stages of kidney function (based on eGFR):**									
≥90 ml/min/$1.73m^2$	1159	Ref		1802	Ref		1954	Ref	
60 to 89 ml/min/$1.73m^2$	1018	-0.81 (-2.07 to 0.45)	0.209	1337	-1.01 (-2.07 to 0.05)	0.062	1456	0.95 (-0.18 to 2.07)	0.100
45 to 59 ml/min/$1.73m^2$	218	-0.08 (-2.19 to 2.04)	0.944	163	-1.99 (-4.50 to 0.51)	0.119	173	0.03 (-2.41 to 2.46)	0.984
<45 ml/min/1.73 m^2	163	-3.94 (-6.36 to -1.52)	**0.001**	77	-3.51 (-6.85 to -0.17)	**0.039**	74	0.46 (-2.69 to 3.61)	0.775
**P trend**			**0.010**			**0.007**			0.329

Abbreviations: CKD: chronic kidney disease; eGFR: estimated glomerular filtration rate, RNFL: retinal nerve fiber layer
†Defined as eGFR < 60 ml/min/ $1.73m^2$
*Adjusted for age, gender, ethnicity, SBP, anti-hypertensive medication, diabetes, hyperlipidemia, body mass index, smoking status, and intraocular pressure
