# Supplementary material for: Associations between Chronic Kidney Disease and Thinning of Neuroretinal Layers in Multiethnic Asian and White Populations
Source: Ophthalmol Sci. 2023 Jun 20;4(1):100353. doi: 10.1016/j.xops.2023.100353 (PMC10587624; doi:10.1016/j.xops.2023.100353)
Supplement: Table S6 [file mmc3.pdf]

Supplementary Table 3. Associations between Chronic Kidney Disease and Kidney Function with Macular GCIPL Thickness by Ethnicity in SEED

	**GCIPL thickness (μm)**								
	**Malay**			**Indian**			**Chinese**		
	**No. of eyes**	**Beta (95% CI)***	**P value**	**No. of Eyes**	**Beta (95% CI)***	**P value**	**No. of eyes**	**Beta (95% CI)***	**P value**
**No CKD**	2057	Ref		2894	Ref		3029	Ref	
**Presence of CKD†**	305	-1.27 (-2.51 to -0.03)	**0.045**	197	-2.07 (-3.53 to -0.62)	**0.005**	179	-1.52 (-3.04 to -0.00)	0.050
**eGFR (Per 10 ml/min/ 1.73m^2 decrease)**	2362	-0.38 (-0.60 to -0.15)	**<0.001**	3091	-0.40 (-0.65 to -0.15)	**0.002**	3208	-0.09 (-0.34 to 0.16)	0.473
**<u>Stages of kidney function (based on eGFR):</u>**									
≥90 ml/min/1.73m^2	1109	Ref		1680	Ref		1768	Ref	
60 to 89 ml/min/1.73m^2	948	-0.82 (-1.65 to -0.00)	0.050	1214	-0.69 (-1.45 to 0.08)	0.077	1261	-0.10 (-0.77 to 0.57)	0.765
45 to 59 ml/min/1.73m^2	175	-0.39 (-1.94 to 1.16)	0.622	133	-2.25 (-4.06 to -0.45)	**0.015**	132	-1.97 (-3.88 to -0.06)	**0.044**
<45 ml/min/1.73 m^2	130	-3.79 (-5.66 to -1.93)	**<0.001**	64	-3.09 (-5.45 to -0.72)	**0.011**	47	-0.66 (-3.10 to 1.78)	0.599
**P trend**			**<0.001**			**<0.001**			0.164

Abbreviations: CKD: chronic kidney disease; eGFR: estimated glomerular filtration rate, GCIPL: ganglion cell-inner plexiform layer
†Defined as eGFR < 60 ml/min/ 1.73m^2
*Adjusted for age, gender, ethnicity, SBP, anti-hypertensive medication, diabetes, hyperlipidemia, body mass index, smoking status, and intraocular pressure
